# Evaluating the Feasibility of an Innovative Self-Confidence Webinar Intervention for Depression in the Workplace: A Proof-of-Concept Study

**DOI:** 10.2196/11401

**Published:** 2019-04-26

**Authors:** Wan Mohd Azam Wan Mohd Yunus, Peter Musiat, June SL Brown

**Affiliations:** 1 Department of Psychology, School of Human Resource Development and Psychology Faculty of Social Sciences and Humanities Universiti Teknologi Malaysia Skudai, Johor Malaysia; 2 Department of Psychology Institute of Psychiatry, Psychology and Neuroscience King's College London London United Kingdom; 3 Department of Psychological Medicine Institute of Psychiatry, Psychology and Neuroscience King's College London London United Kingdom

**Keywords:** online, videoconferencing, cognitive behavioral therapy, depression, workplace

## Abstract

**Background:**

Depression in the workplace is a very common problem that exacerbates employees’ functioning and consequently influences the productivity of organizations. Despite the commonness of the problem and the currently available interventions, a high proportion of employees do not seek help. A new intervention, a webinar (Web-based seminar), was developed, which integrated the use of technology and the traditional guided therapist support to provide accessible help for the problem of depression in the workplace.

**Objective:**

The aim of this study was to explore the feasibility, preliminary outcome, and acceptability of the webinar intervention conducted in organizations.

**Methods:**

In total, 2 organizations were invited to participate, and 33 employees participated in this proof-of-concept study. The webinar intervention consisted of 6 1-hour sessions conducted via the Adobe Connect platform, developed by Adobe Inc. The intervention was developed based on a systematic review, focus group studies, and face-to-face self-confidence workshops that utilized cognitive behavior therapy (CBT). The final webinar intervention used CBT and the coping flexibility approach. The structure of the intervention included PowerPoint presentations, animation videos, utilization of chat panels, and whiteboard features. The intervention was conducted live and guided by a consultant psychologist assisted by a moderator. Study outcomes were self-assessed using self-reported Web surveys. The acceptability of the intervention was assessed using self-reported user experience Web surveys and open-ended questions.

**Results:**

The findings showed: (1) evidence of feasibility of the intervention: the webinar intervention was successfully conducted in 3 groups, with 6 1-hour sessions for each group, with 82% (23/28) participants completing all 6 sessions; (2) positive improvements in depression: the linear mixed effects modeling analysis recorded a significant overall effect of time primarily for depression (*F*_2, 48.813_=31.524; *P*<.001) with a Hedge g effect size of 0.522 at 1-month follow-up. Individually, 8 subjects showed significant reliable and clinically significant changes, with 3 subjects showing clinically significant change only; and (3) encouraging evidence regarding the acceptability of the webinar intervention among the employees: the user experience score was above average for 4 out of 6 domains measured (perspicuity mean 1.198 [95% CI 0.832-1.564], efficiency mean 1.000 [95% CI 0.571-1.429], dependability mean 1.208 [95% CI 0.899-1.517], and stimulation mean 1.323 [95% CI 0.987-1.659]). The open-ended questions also yielded 52% (47/91) of the responses that reported facilitators, whereas only 12% (11/91) of the responses reported barriers.

**Conclusions:**

The self-confidence webinar intervention appears to be a potentially feasible, effective, and acceptable intervention for depression in the workplace that merits further investigation.

## Introduction

### Background

Depression has been shown to cost employers in Europe £77 billion each year [1]. However, despite the high prevalence of depression, employees are reluctant to seek help. One possible reason for this is that employees view seeking help as a sign of weakness rather than a medical condition that can be treated [2]. Obstacles to seeking help can also be seen in the general public. The general public often conceptualizes depressive symptoms as *problems of living* rather than symptoms of a mental illness [3]. Recent studies have found that interventions that used nondiagnostic labels such as *self-confidence* rather than depression are important in engaging groups who may prefer not to medicalize mental health [4,5]. This also provides an alternative and accessible route to psychological help, which is more congruent with the health beliefs of the public [5]. This self-confidence program has also been shown to maintain effects after 2 years [6].

Self-confidence workshops using a self-referral system, which were designed to be accessible to adults with depression in the community, found the workshops to be clinically effective and reached a large number of people who were reluctant to engage [5]. However, participants were largely not employed [5,7]. This suggested that a different approach would be needed for the workplace. It has been suggested that the workplace is an ideal setting to provide information about depression to employees [2].

A systematic review by Wan Mohd Yunus et al [8] reported that most evidence-based workplace interventions for depression were delivered face-to-face either individually or in groups. However, more recently, technological advances have been used in the mental health services and showed promising results, using methods such as interventions on the internet, software packages, and mobile apps. The integration of therapist support with technology-mediated interventions could also have a strong influence on new intervention processes and outcomes. It is likely that these technological advances could improve access and have other advantages such as reduced stigma, facilitating some people to share about sensitive issues, shortening waiting lists and cost-effectiveness [9]. Despite these available interventions, mental health services are still underutilized by employees [10,11].

Given the technological advances, the importance of depression in the workplace and the problems in reaching people with depression who are employed, a more interactive form of computerized cognitive behavior therapy (CBT) using a webinar (a seminar delivered over the Web) was developed. Using the self-confidence label to make the intervention more accessible to people with depression, the webinar aimed to combine aspects of traditional face-to-face interventions and also take advantage of the technology that is currently available to develop an innovative webinar package for reducing depression in the workplace.

The intervention development process was in line with the Medical Research Council guideline for developing and evaluating complex interventions [12]. It involves a systematic review [8] and results from focus groups [13], as well as previous research by Brown et al [5,6,14,15] and Cheng et al [16,17]. The new intervention incorporates a self-confidence webinar delivered during working hours in the workplace and is, to our knowledge, the first webinar intervention developed to target depression in the workplace. This webinar also used the more *user-friendly* and nondiagnostic label of self-confidence to reach out to more people, especially to those who were previously reluctant to seek help [4,5,15]. This research reports on the preliminary findings of this new webinar intervention regarding its feasibility, preliminary outcomes, and acceptability, which was tested pragmatically with interested organizations.

### Aim

The aim of this study was to assess feasibility, preliminary outcomes, and acceptability of running the self-confidence webinar in the workplace. This is in line with Eldridge et al’s recommendation that feasibility studies should include *studies assessing whether a future study, project or, development can be done* [18].

### Objectives

The first objective of this study was to assess whether the self-confidence webinar was feasible and whether the intervention could be implemented as planned. It was decided that the intervention would be feasible if these objectives were met:

1. At least 20 employees were recruited;
2. A total of 6 sessions were able to be conducted;
3. Dropout rates were below 32%;
4. Completion rates were at least 80% (participants attending all 6 sessions);
5. There were no major technical issues that significantly affected the running of each session.

The second objective was to investigate the preliminary outcomes of a self-confidence webinar to reduce depression and to investigate whether there was an improvement in other measures including anxiety, self-esteem, coping flexibility, absenteeism, and presenteeism. The third objective was to investigate the acceptability of the intervention and whether participants were satisfied with the intervention.

## Methods

### Design

This proof-of-concept study employs a single-group pre-post design. Assessments were taken at baseline, postintervention, and 1 month postintervention. The study was granted ethical approval by the King’s College London Research Ethics Committee (LRS-14/15-0857).

### Procedure of Recruiting Organizations and Participants

The recruitment processes involved several stages: (1) Identifying interested organizations; (2) initial communication with interested organizations; (3) meeting with line managers and well-being teams to obtain permission from the organization; (4) if the organization was interested, on-site testing of technical aspects of readiness for the webinar was conducted; (5) organizations were provided with information about the study, which was then disseminated to the organizations’ employees. Employees thus received flyers and an information sheet about the webinar and research from the gatekeeper (the lead person from each organization). The information sheet included detailed information about the study, whereas the flyer included an invitation to the introductory meeting; and (6) If any employees were interested, they were asked to contact the first author and were invited to the introductory meeting held in an office in the organization. Alternatively, if they were unable to attend the introductory meeting, interested employees were provided with further information about the study. To maintain confidentiality, employees’ managers were not informed about which employees had participated.

Before the webinar, consent from line managers or team leaders was obtained as the sessions were to be conducted during working hours, as this was a strong view from the focus group that was conducted before the webinar was offered [13].

### Intervention

The Template for Intervention Description and Replication checklist and guide was employed to describe the intervention [19]. The self-confidence workshop program was offered as Web-based seminars using a Web-based webinar provider (Adobe Connect) with the participating employees. The webinar intervention for improving self-confidence lasted 6 weeks, with 1 session per week. Every session involved an hour-long live webinar, which included time for questions as well as homework. Each session involved a PowerPoint presentation with videos and comic strips, an *Interactive Zones* component where participants interacted with each other, a virtual whiteboard, use of the chat feature, and a webcam feature for the therapist. To attend the sessions, participants could use a computer, laptop, or mobile device including smartphones (iPhones or Android phones) and tablets (iPads and tablets). Thus, attendance at these sessions was possible wherever the internet was available. The content of the sessions was based on a cognitive-behavioral approach by Brown et al [5,6,14,15] and coping flexibility by Cheng et al [17] and is outlined in Textbox 1. Multimedia Appendix 1 includes screenshots of the webinar sessions.

### Measures

#### Depression

The Beck Depression Inventory II (BDI-II) is a widely recognized 21-item self-report inventory for the assessment of depressive symptoms. Each item consists of 4 statements scored on a scale ranging from 0 to 3 with higher scores indicating higher depressive symptoms. The scores can be calculated and categorized into 4 levels of severity: minimal (0 to 13); mild (14 to 19); moderate (20 to 28); and severe (29 to 63) [20].

**Main elements of each session.**

Session 1: Introduction to self-confidence in the workplace

- Introduction to the whole program; self-confidence and challenges at work; the self-confidence model; how low self-confidence develops

Session 2: Thinking differently

- Negative automatic thoughts; understanding unhelpful thinking beliefs; identifying and challenging negative thoughts; distraction, thought stopping, and coping self-statements

Session 3: Changing our self-image

- Development of poor self-image; changing our self-image; what can influence my self-image; anxiety and performance

Session 4: What you can do (Part 1)

- Behavior and self-confidence; problem-solving skills; managing time effectively; brief relaxation

Session 5: What you can do (Part 2)

- Assertiveness; our support system; making change happen; setting our goals

Session 6: Being more flexible

- Coping flexibility concept; the process of coping flexibly; how to use coping strategies flexibly; case study

#### Anxiety

The Beck Anxiety Inventory is a 21-item self-report inventory to assess overall anxiety. Respondents are asked to rate the severity of each symptom using a 4-point scale with higher scores reflecting higher anxiety. The sum of all items corresponds to 4 levels of severity: minimal anxiety (0 to 9); mild anxiety (10 to 16); moderate anxiety (17 to 29); and severe anxiety (30 to 63) [21].

#### Self-Esteem

The Rosenberg Self-Esteem Scale is a 10-item self-report inventory to measure individual self-esteem. Each item comprises a 4-point scale which contains 5 positively and 5 negatively worded items. The scores of all items are totaled with a higher score suggesting a higher level of self-esteem [22].

#### Coping Flexibility

Coping flexibility was measured using the 10-item Coping Flexibility Scale. This self-report inventory measures an individual’s ability to evaluate coping strategies and adopt alternative coping strategies depending on the situation. Respondents are required to respond to the items on a 4-point scale, and the sum of all items represents the overall score of coping flexibility with a higher score indicating a higher level of coping flexibility [23].

#### Presenteeism and Absenteeism

The short form World Health Organization Health and Work Performance Questionnaire is a self-report inventory that measures absenteeism and presenteeism. For absenteeism, respondents respond to 8 items that relate to the number of hours lost per month, with a higher score indicating a higher amount of absenteeism. Absolute absenteeism is measured in raw hours where a negative lower bound suggests that employees work more than expected and a higher score indicates a higher amount of absenteeism. Relative absenteeism is expressed as the percentage of expected hours whereby a negative number suggests that individuals work more than expected, and a score of 1.0 suggests that the individual is always absent [24]. There are 3 items for presenteeism that correspond to reduced work performance with a higher score indicating a lower amount of lost performance. An absolute presenteeism score with a lower bound of 0 suggests a total lack of performance during the time on the job, whereas an upper bound of 100 indicates no lack of performance during the time on the job. For relative presenteeism, a minimum score of 0.25 indicates the worst relative performance compared with other employees, whereas a maximum score of 2.0 indicates the best performance compared with other employees [24].

#### User Experience Questionnaire for Acceptability Measure

The User Experience Questionnaire (UEQ) is a self-report inventory to assess the ability of a product to engage the user. It consists of 26 items grouped by 6 domains: Attractiveness, Efficiency, Perspicuity, Dependability, Stimulation, and Novelty. Each item is randomly ordered along a 7-point scale representing 2 graded contrasting attributes [25]. Values between –0.8 and 0.8 represent a neutral evaluation of the corresponding scale. On the contrary, values >0.8 represent a positive evaluation, whereas values <–0.8 represent a negative evaluation. Although the range of the scale is between –3 (horribly bad) and +3 (extremely good), it is extremely unlikely to observe values above +2 or below –2 [26].

#### Open-Ended Satisfaction Questions

Additionally, 4 open-ended questions were also included in the postintervention Web-based questionnaire. The questions were as follows:

1. “If the webinar was to run again, can you please recommend things we should start doing that we are not currently doing?”
2. “What are the things we should stop doing that we did during this webinar that did not work for you?”
3. “What are the things that worked well that we should continue doing?”
4. “Please type in the space below if you have any other comments/feedback or suggestions about the webinar program.”

### Data Analysis for Outcome Measures

Descriptive statistics were used to report the scores at baseline (T_0_), postintervention (T_1_), and 1-month postintervention (T_2_). The data were then analyzed using a mixed model analysis or a nonparametric Friedman test to analyze the difference in scores at T_0_, T_1_, and T_2_. For linear mixed effects modeling, the required assumptions were initially met for self-esteem and coping flexibility. Attempts to transform the data were performed on other variables but were only successful for depression and anxiety by using square root transformation. For absolute absenteeism, relative absenteeism, absolute presenteeism, and relative presenteeism variables, the nonparametric Friedman test was performed.

Effect sizes and reliable and clinically significant changes were computed using data from completers at 1-month follow-up as well as those who only provided postintervention data but did not respond to the follow-up. Postintervention data were used instead if the follow-up data were not available.

The standardized effect size was calculated using both Cohen *d*_av_ and Hedge g correction. Lakens [27] recommended the use of Cohen *d*_av_ for within-subject research design but because this is positively biased as it is based on a sample estimate, Hedges g_av_ correction was also applied. The effect size (Hedge g_av_) of the change in scores was calculated manually using a scientific calculator based on the following formula:

Hedges g_av_=M_diff_/[(SD_1_ + SD_2_)/2] x [1–3/[4(n_1_ + n_2_)–9]

The reliable and clinically significant changes for depression were calculated using the following formulas [28-30]:

Reliable Change Index (RCI)=√(2 [SD_pre_ x √(1–α)]^2^) x 1.96

Clinically significant change=[(mean_clin_ x SD_norm_) + (mean_norm_ x SD_clin_)]/(SD_norm_ + SD_clin_)

The number of participants and their depression level classification at each time point are also described.

## Results

### Participating Organizations

In total, 2 participating organizations were recruited. One organization was an administrative body funded directly by the government. The other organization was a local authority of one of the 32 London borough councils in the United Kingdom.

### Participants

In total, 33 employees volunteered to participate. Table 1 displays the demographic characteristics of the study participants.

### Objective 1: Feasibility of the Self-Confidence Webinar

All feasibility criteria were met, indicating that the self-confidence webinar was feasible for a larger study in the future. This study showed that:

1. 33 employees were recruited;
2. The webinar was successfully conducted for 3 groups, with 6 sessions for each group;
3. The dropout rate was 24% (8/33);
4. All 6 sessions were completed by 82% (23/28) participants;
5. There were no major technical issues that significantly affected the running of each session.

The initial list consisted of 6 organizations, where interest from these organizations was initially established from previous research or contact involving the third author. However, following initial contact, 2 organizations did not follow up their interest and another 2 chose to opt out because of practicality (confidentiality and open plan office) and information technology resource issues. In total, 2 organizations decided to proceed with the next stage.

**Figure 1:**
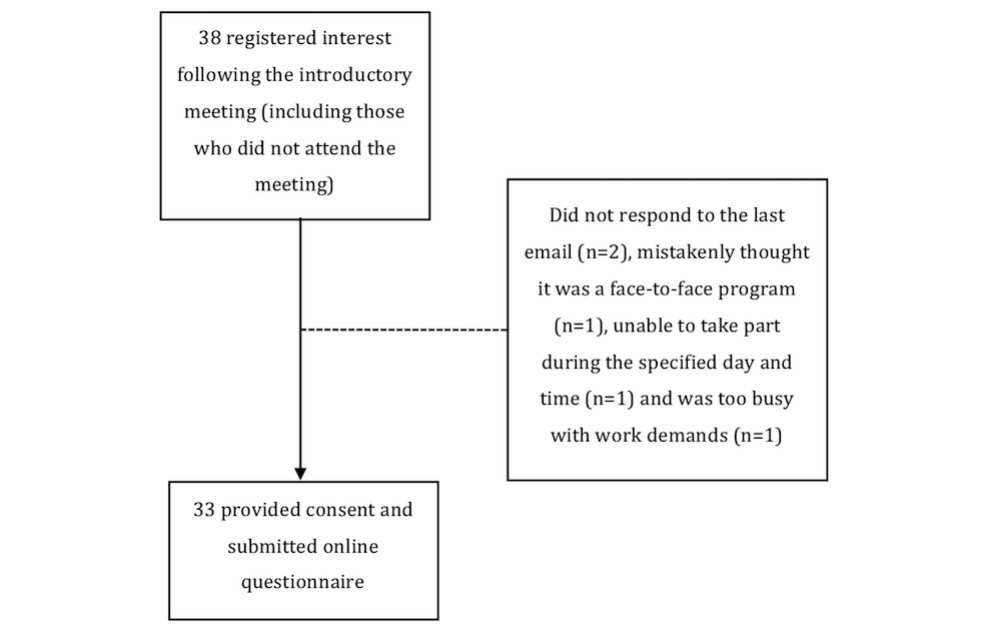
Summary of the recruitment process.

A total of 37 employees attended the on-site introductory meetings. There were also some employees who contacted the first author directly for further questions and/or registered their interest but did not come to the meeting. Overall, there were 38 employees who attended or contacted the first author directly who ended up registering their interest with the webinar. Of the 38 employees, 33 provided consent and submitted the Web-based baseline questionnaire, following ongoing email and telephone communications with the first author. Reasons for nontakeup included 2 who did not respond to the invitation email, 1 mistakenly thinking it was a face-to-face program, 1 being unable to take part during the specified day and time, and 1 being too busy with work demands. Figure 1 displays the summary of the recruitment and reach process.

**Table 1:** Demographic characteristics (n=33).

Demographics		Statistics
Age (years), mean (range)		39.6 (21-62)
**Gender, mean (SD)**		
	Female	30 (90.9)
	Male	3 (9.1)
**Marital status, mean (SD)**		
	Single	12 (36.4)
	Married or civil partnership	10 (30.3)
	Living together	9 (27.3)
	Divorced	2 (6.1)
**Highest level of education, mean (SD)**		
	University degree	17 (51.5)
	Postgraduate degree	6 (18.1)
	A level or National Vocational Qualification	5 (15.2)
	Diploma or Business and Technology Education Council	5 (15.2)
**Ethnicity, mean (SD)**		
	White—English or Welsh or Scottish or Northern Irish or British or Irish or any other white background	18 (54.5)
	Black or African or Caribbean or black British—Caribbean or African	6 (18.2)
	Asian or Asian British—Indian or Bangladeshi or any other Asian group	6 (18.2)
	Mixed or multiple ethnic groups—white and black Caribbean or white and black African	3 (9.1)
Work experience (years), median (range)		18 (2-45)

In total, 2 organizations participated and each held one introductory session. Owing to the timing preferences of the employees, 3 webinar groups were held for 33 participants. The webinar presenter and first author, who acted as the moderator, conducted all 6 sessions of the webinar, giving a total of 18 sessions conducted for the 3 groups.

Of the 33 participants who initially registered, 28 took part in the first session and 25 took part in the sixth session, meaning 8 participants dropped out (24%). However, if only those who attended session 1 are included, the dropout rate was only 11% (3/28). Notably, 82% (23/28) participants completed all 6 sessions and 86% (24/28) participants completed at least 4 sessions. The reasons for not being able to attend the live session included attending other meetings and being on leave. The follow-ups at postintervention and at 1-month postintervention were conducted over the Web and blind-carbon copies, with reminder emails, were also sent intermittently. A total of 73% (24/33) postintervention questionnaires were obtained at postintervention and 70% (23/33) questionnaires obtained at 1-month follow-up.

### Objective 2: Preliminary Outcomes of the Self-Confidence Webinar

#### Descriptive Data

Table 2 shows the means and SDs of the outcome measure scores. Depression, anxiety, absolute absenteeism, and relative absenteeism decreased at T_1_ and T_2_, whereas self-esteem, coping flexibility, absolute presenteeism, and relative presenteeism increased at T_1_ and T_2_.

**Table 2:** Means and SDs of outcome measure scores. T_0_: baseline; T_1_: postintervention; T_2_: 1-month postintervention.

Measures	T_0_ (n=33), mean (SD)	T_1_ (n=24^a^), mean (SD)	T_2_ (n=23^a^), mean (SD)
Depression	13.91 (9.77)	8.63 (8.71)	9.09 (8.47)
Anxiety	11.42 (9.17)	10.42 (10.98)	9.09 (9.96)
Self-esteem	18.15 (4.87)	19.63 (5.75)	20.09 (5.67)
Coping flexibility	14.27 (6.17)	18.42 (4.93)	18.96 (5.14)
Absolute absenteeism	8.52 (27.51)	1.54 (17.30)	5.20 (25.58)
Relative absenteeism	0.06 (0.20)	0.02 (0.15)	0.03 (0.18)
Absolute presenteeism	57.58 (14.15)	69.58 (15.46)	71.30 (13.92)
Relative presenteeism	0.78 (0.18)	1.01 (0.23)	1.19 (0.65)

^a^Only available data are included.

#### Linear Mixed Effects Modeling

To get a preliminary estimate of the improvement in the outcome measures in a group of participants for the webinar intervention, linear mixed effect modeling was conducted. Time of assessment (T_0_, T_1_, and T_2_) was included as a main effect in the fixed factors of the model to account for the 3 different time points of the assessment. A random intercept by participants was included in the model to account for the different scores at baseline. An autoregressive -AR(1) covariance matrix was used to account for constant variance at each time point assuming that the correlation gets less as time points get further apart (T_0_, T_1_, and T_2_). Maximum Likelihood estimation was therefore chosen to fit the models.

The overall effect of time was significant for all measures: depression (*F*_2, 48.813_=31.524; *P*<.001), anxiety (*F*_2, 49.428_=3.945; *P*=.03), self-esteem (*F*_2, 49.519_=31.524; *P*=.02), and coping flexibility (*F*_2, 48.623_=14.184; *P*<.001). Table 3 shows the summary of the result of the mixed models analyses for depression, anxiety, self-esteem, and coping flexibility.

**Table 3:** Pairwise comparison from linear mixed effects modeling analysis. T_0_: baseline; T_1_: postintervention; T_2_: 1-month postintervention.

Time point	Depression^a^		Anxiety^a^		Self-esteem		Coping flexibility	
	Mean (95% CI)	*P* value	Mean (95% CI)	*P* value	Mean (95% CI)	*P* value	Mean (95% CI)	*P* value
T_0_	3.492 (3.030-3.954)	—^b^	3.064 (2.529-3.599)	—	18.152 (16.375-19.928)	—	14.273 (12.371-16.175)	—
T_1_	2.400 (1.920-2.880)	—	2.717 (2.159-3.276)	—	19.510 (17.651-21.369)	—	18.550 (16.487-20.613)	—
T_2_	2.514 (2.001-2.993)	—	2.415 (1.836-2.994)	—	20.324 (18.394-22.254)	—	19.380 (17.217-21.542)	—
T_0_ versus T_1_	1.092 (0.751 to 1.433)^c,d^	<.001	0.346 (–0.078 to 0.770)^c,d^	.14	–1.359 (–2.813 to 0.095)^c,d^	.07	–4.277 (–6.434 to –2.120)^c,d^	<.001
T_0_ versus T_2_	0.995 (0.530 to 1.460)^c,d^	<.001	0.649 (0.075 to 1.223)^c,d^	.02	–2.173 (–4.133 to –0.212)^c,d^	.02	–5.107 (–7.861 to –2.353)^c,d^	<.001

^a^Due to non-normality of the residuals, square root transformation was performed.

^b^—: not applicable.

^c^Significance level *P*<.05.

^d^M_diff_ (95% CI).

#### Friedman Tests

Both absolute and relative absenteeism were maintained throughout the 3 time points at T_0_, T_1_, and T_2_ (median=0), and the differences were not statistically significant (absolute absenteeism, χ^2^_2_=2.358; *P*=.31; Relative absenteeism, χ^2^_2_=1.701; *P*=.43).

For presenteeism outcomes, pairwise comparisons were performed with a Bonferroni correction for multiple comparisons. Absolute presenteeism was statistically significantly different at the different time points during the study period; χ^2^_2_=22.116; *P*<.001. A posthoc analysis revealed statistically significant differences in absolute presenteeism from T_0_ (median=60.00) to T_1_ (median=70.00; *P*=.006) and T_0_ to T_2_ (median=80.00; *P*<.001). Relative presenteeism was statistically significantly different at the different time points during the study period; χ^2^_2_=26.325; *P*<.001. A posthoc analysis revealed statistically significant differences in absolute presenteeism from T_0_ (median=0.750) to T_1_ (median=1.000; *P*=.002) and T_0_ to T_2_ (median=1.125; *P*<.001).

#### Effect Sizes

The results are summarized in Table 4. Overall, improvements were recorded for all measures at T_1_ and T_2_ when compared with T_0_ with the highest effect size recorded for presenteeism measures, followed by coping flexibility and depression. The positive Hedges g_av_ indicated a reduction in scores, whereas the negative Hedges g_av_ indicated an increase in scores.

#### Reliable Change

The reliable and clinically significant change analysis only includes participants who completed the assessment at all 3 time points or those who completed the assessment at T_0_ and T_1_ or T_2_. The SD of the BDI-II at baseline (SD_pre_) was 10.18, whereas the calculated Cronbach alpha was .92. The calculated RCI was 7.98. Thus, the changes in BDI-II scores can be categorized into 3 groups: a reliable increase in depression symptoms (an increase of 8 points more on the BDI-II), no reliable change (less than 8 points increase or decrease on the BDI-II), and reliable improvement in depression symptoms (a reduction of 8 points or more on the BDI-II). On the basis of the data in Table 5, a total of 35% (9/26) participants showed reliable change and a reduction in depression symptoms. In contrast, 65% (17/26) participants showed a change smaller than this.

**Table 4:** Hedges g_av_ effect sizes of outcome measure scores. T_0_: baseline; T_1_: postintervention; T_2_: 1-month postintervention.

Measures	Hedges g_av_	
	T_0_ versus T_1_	T_0_ versus T_2_
Depression	0.563	0.522
Anxiety	0.098	0.240
Self-esteem	–0.275	–0.363
Coping flexibility	–0.738	–0.817
Absolute absenteeism	0.307	0.123
Relative absenteeism	0.219	0.156
Absolute presenteeism	–0.799	–0.963
Relative presenteeism	–1.107	–0.962

**Table 5:** Cross tabulation summary of reliable change against clinically significant change.

Clinically significant change (score criterion of >12)	Total at baseline	Reliable change postintervention (change of score of >8)	
		Yes	No
Started lower than the criterion for clinically significant change (baseline score <12)	10	0	10
Started higher than the criterion but failed to achieve clinically significant change (baseline score >12 but postintervention score still >12)	5	1	4
Clinically significant change (baseline score >12; postintervention score <12)	11	8	3
Total	26	9	17

#### Clinically Significant Change

To calculate this change, normative data from a community sample of 7500 respondents in another European study using BDI-II were used, which showed a mean score of 10.6 and an SD of 10.9 [31]. Hence, using the formula in the Methods section, the clinically significant change criterion score was calculated to be 12.34. In summary, 38% (10/26) participants started with baseline BDI-II scores below the criterion and therefore did not show clinically significant change and none showed reliable improvement. In total, 19% (5/26) participants started with baseline scores above the criterion but failed to improve to a lower score below the criterion, with 4% (1/26) participant showing reliable improvement. In total, 12% (3/26) participants did not record reliable change despite recording clinically significant change. This suggests that it is not sufficient to imply that the change is not influenced by simple measurement unreliability [28]. Finally, 42% (11/26) participants showed clinically significant change, of whom 31% (8/26) also showed reliable improvement. Table 5 summarizes the data for reliable and clinically significant change.

#### Depression Level at Different Time Points

**Figure 2:**
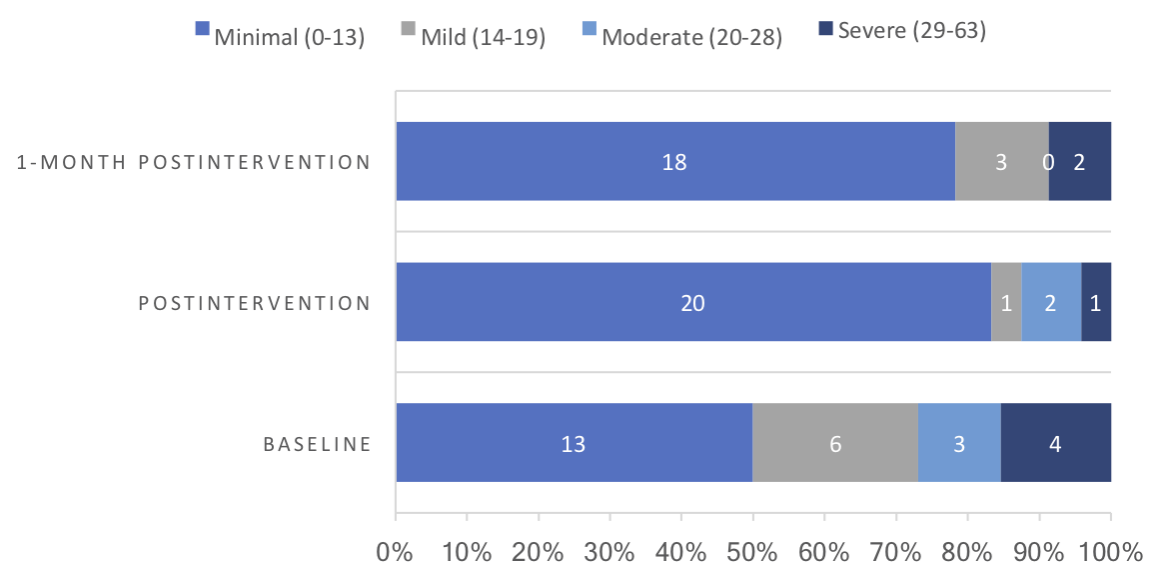
Distribution of scores for depression.

The distribution of scores for depression at each time point is presented in Figure 2. The figure only includes available data at the 3 time points. At baseline, nearly a quarter of the participants had a moderate-to-severe level of depression symptoms. Generally, improvement was observed in terms of the depression severity when compared with baseline. The percentage of employees in moderate and severe depression severity decreased when compared with baseline.

### Objective 3: Acceptability of the Self-Confidence Webinar

#### User Experience

On the basis of the result of the UEQ, participants reported satisfaction in all domains: Attractiveness, Perspicuity, Efficiency, Dependability, Stimulation, and Novelty.

Although figures are not available for comparing the webinar intervention with any other type of psychological intervention, the UEQ is able to compare the measured user experience with the results from 163 different types of products such as business software, Web pages, Web shops, and social networks with a total of 4818 participants [26]. Table 6 displays the user experience results and the interpretation of the results of the webinar intervention compared with the user experience of other products.

**Table 6:** Interpretation of the webinar intervention in comparison with other interactive products.

Scale	Mean (95% CI)	Relative comparison to other products	Interpretation
Attractiveness	1.618 (1.242-1.994)	Good	10% of results better; 75% of results worse
Perspicuity	1.198 (0.832-1.564)	Above average	25% of results better; 50% of results worse
Efficiency	1.00 (0.571-1.429)	Above average	25% of results better; 50% of results worse
Dependability	1.208 (0.899-1.517)	Above average	25% of results better; 50% of results worse
Stimulation	1.323 (0.987-1.659)	Above average	10% of results better; 75% of results worse
Novelty	1.083 (0.718-1.449)	Good	10% of results better; 75% of results worse

#### Open-Ended Questions

Facilitators, barriers, suggestions, and general comments were analyzed separately using qualitative content analysis on each question using Microsoft Excel. As this was likely to be the first study exploring the application of a webinar intervention within the workplace setting, an inductive approach was chosen [32]. The responses were divided into 4 main categories: facilitators 52% (47 out of 91), barriers 12% (11 out of 91), suggestions 14% (13 out of 91), and general comments 22% (20 out of 91). Example quotes from the feedback corresponding to each category are presented in the Multimedia Appendix 2.

## Discussion

### Principal Findings

The findings of this study indicate that the innovative self-confidence webinar intervention is feasible, and positive improvements were observed particularly on depression, presenteeism, and other outcome measures. Moreover, employees also reported that the webinar intervention was very acceptable.

### Feasibility

The webinar recorded low dropout rates and high completion rates among employees who registered. Although the sample size was small, the self-confidence webinars also had low dropout rates with high completion rates among those who attended the first session. Of the 33 registered participants, 25 (76%) completed the final session, giving a dropout rate of 24%. Of the 85% (28/33) participants who attended the first session, 82% (23/28) completed all 6 sessions, giving a dropout rate of 18%. These figures are favorable than the completion rates (66%) in a much larger multicenter randomized controlled trial (RCT) of face-to-face psychoeducational CBT workshops [5] and multiservice practice research using the Stress Control (SC) program that recorded a completion rate of 70% [33]. The SC program study highlights the importance of participants’ subsequent attendance as one of the predictors for a positive outcome.

There is much evidence of the high dropout rate for computerized CBT. A systematic review on barriers and uptake of computerized CBT reported that those receiving computerized CBT were twice as likely to drop out as those receiving face-to-face CBT [34]. Another meta-analysis on computer-based psychological treatment for depression however found a variation and reported a dropout rate of 28% for interventions with therapist support, 38.4% for administrative support, and 74% for no support [35]. The dropout rate of 24% found in this study is therefore comparable with that of supported psychological therapies.

The fact that the presenter and moderator played significant roles in the webinar throughout all sessions indicates that it was a highly guided intervention. Hence, the dropout rates and completion rates among the webinar participants were comparable with other guided internet interventions. A meta-analysis review of the dropout rates for guided internet interventions compared with face-to-face interventions showed no significant difference [36]. Moreover, the percentage of completed sessions by the subjects was also found to be comparable between guided internet-based interventions and face-to-face interventions for depression. A study reported that the mean completion rate for face-to-face CBT was 83.9%, whereas the mean completion rate for guided internet CBT was 80.8% [37]. The review highlights the importance of the guided component within a technology-mediated intervention.

It is also important to note that the self-confidence webinar was accessed by employees in different age ranges (range=21-62; mean 39.6), as age has been found to be a highly significant factor in accessibility and utilization of the internet and new technology [38]. It has been argued that the use of the internet falls off sharply with age, owing to factors such as attitudinal beliefs, age-related changes, cognitive barriers, and privacy concerns [39]. It was not found to be the case with the webinar.

However, there are several challenges that need to be highlighted. First, the difficulties in the recruitment process of organizations need to be mentioned. One organization had undergone restructuring during the study period, which involved the movement of employees, which in turn affected the communication between the first author and the organization. Olsen et al [40] highlighted these challenges and suggested that changes in the organizations should be considered when planning intervention studies; this, in turn, can cause delays and communication problems with the research.

Another aspect was the competing time commitments of employees. Owing to the live nature of the webinar, employees are required to access the webinar intervention during a predetermined date and time. Although recorded versions of the webinar were available, it was felt that the learning experience and contribution of participants would be more restricted than attending live sessions. When this problem did occur, employees were provided with the full recording of that particular webinar session. With this, they were able to follow the session asynchronously and obtain some input about the session.

Technical barriers to accessing the webinar program may be important. For instance, in one of the organizations, there was an initial difficulty in accessing the webinar program because of the tight internet security settings used by the organization. This was used to restrict the use of internet connection to only Web pages and links that were directly related to work. Although this issue was successfully overcome, it is important for future research to take note of this problem and make sure that on-site testing is conducted before the intervention is implemented. Furthermore, it is important to make sure that the computer, laptop, or mobile devices fulfill the minimum technical specification required to run the webinar program.

### Outcomes

The follow-up results after 1 month suggested positive changes for all outcome measures. For depression, the analysis indicated that symptoms of depression significantly improved at postintervention and 1 month postintervention compared with baseline with moderate effect sizes of 0.563 and 0.522, respectively. This result was lower than the 0.844 effect size obtained in the RCT study of group face-to-face self-confidence workshops to reduce depression for the public [5]. Nevertheless, the outcome of this webinar study suggested that adaptations to the intervention content, through a webinar platform, produce encouraging results for the intervention in the workplace, especially considering that this is a universal intervention. This small pre-post study provides preliminary support for the position that a guided webinar form of delivery of an intervention can provide positive effects for individuals.

Notably, although the intervention was presented as *self-confidence* and targeted self-esteem, it was interesting that the difference in effect sizes for self-esteem was less significant than that of depression with recorded effect sizes of 0.563 postintervention and 0.522 at follow-up for depression and –0.275 postintervention and –0.363 at follow-up for self-esteem. More importantly, this may suggest that the use of a nondiagnostic label of self-confidence, compared with the more medicalized label of *depression*, was successful in attracting people with some degree of depression. Consistent with other studies, the use of a diagnostic label, such as *depression* or *insomnia*, may affect the uptake and engagement of intervention with people who would prefer not to medicalize mental health [4,5,15].

An important finding from this study was that the largest effect size found at 1 month postintervention was for the outcome measurement of presenteeism (absolute presenteeism=–0.963 and relative presenteeism=–0.962). This is a significant finding as the intervention contributes a positive impact to work-related outcomes such as presenteeism.

Although the positive outcomes may be contributed to by intervention content, it is also important to highlight the *group* nature of the webinar. During each session, participants interacted with other participants, as well as the webinar presenter; this was especially the case during the *interactive zones*. Previous studies have provided the benefits of group intervention, such as providing natural social networks, being cost-effective and more accessible, providing opportunities to learn and support others, having less stigma, and obtaining more neutral views from other participants rather than the therapist, as well as being useful for those who are uncomfortable or struggle with individual intervention [41-44].

Owing to the universal nature of the intervention, it was expected that participants would consist of those with mixed levels of self-reported depression. It follows that there were participants with few or no depressive symptoms reported at baseline as well as participants with varying depressive severity levels, supporting the findings from a systematic review that universal interventions also attract those with higher levels of depression [8]. The mean score for depression among the participants was 13.91 on the BDI-II, indicating mild depression. More importantly, in line with previous self-confidence workshops that used a self-referral system and where 75% met diagnostic levels of problems [7], the webinar intervention also attracted those with higher levels of depression and not just those who are *worried well*.

### Acceptability of the Self-Confidence Webinar

The majority of webinar participants gave positive and encouraging feedback about the acceptability of the self-confidence webinar. In terms of user experience, participants indicated that they really liked the webinar and found it to be attractive. In line with Daft and Lengel’s Media Richness Theory [45], the webinar can be considered as a *rich* medium as it utilizes a number of different channels and cues to enhance the effectiveness of the communication. The participants also reported it to be exciting and motivating, suggesting most stayed throughout the program. Additionally, they also found it easy to use, felt in control, and able to use the webinar effortlessly, as well as considered it innovative and creative.

Furthermore, positive facilitator factors were identified; the *interactive zones* and content recorded the highest number of positive feedback comments. The webinar consists of 2 main components: visual (eg, share visual files, webcam, and virtual whiteboard) and audio (eg, telephone or voice over internet protocol) components that provide different ways to facilitate interaction [46]. It is reassuring that participants found the content and intervention to be useful for them, indicating that the material had been well-adapted for this purpose. Most of the positive feedback was related to the implementation of the *interactive zones*, which was the main adaptation made to allow engagement between participants and the webinar’s presenter. This is encouraging, as this finding suggests that the application of a Web-based interaction platform through the webinar was well-received by the intervention participants. Previous evidence has also reported that webinars with its interactive features may facilitate participant interaction while being capable of providing a communication environment that is nearly the same as a face-to-face environment [47]. Notably, an important element of the webinar is the presence of a therapist and moderator. Moreover, with consent from the participants, each session was also recorded, and the participants could access the session anywhere and anytime. These benefits are in line with the findings from a Delphi study that highlighted the importance of effective moderation within an online discussion forum and 24-hour mobile access for the design of a Web-based intervention [48].

An important feature of the webinar that emerged during the study was that it can be accessible without restricting geographical context. In this study, a participant reported attending a session from abroad during a 3-week holiday. Another participant also reported attending a session at home while on leave rather than at work. Even with limited evidence, this study showed that some of the barriers to accessing treatment, which may be present in normal face-to-face intervention, were overcome in the webinar intervention.

### Limitations and Future Directions

There were several limitations of this study. First, the timeframe and nature of the recruitment process made it possible to only have an intervention group with no control group. The absence of a control group means it is not possible to determine whether the improvement in outcome scores is because of the intervention or other factors not covered in the study, such as time or a placebo effect. Second, the small sample size limits the ability to generalize from these results about other employees in other organizations. Hence, the results and conclusions derived from this study need to be interpreted with caution. Third, the unbalanced number of male and female participants may limit the extent to which the results can be generalized to males. Although this is a common pattern among mental health intervention studies, it needs to be noted. Fourth, all outcome measures were based on self-reports, which limits the quality of the data; this was the case for the absenteeism measure. Access to data on absenteeism via the participating organizations was not planned nor deemed feasible within the research timescale. Finally, the study only utilizes a short follow-up (1 month postintervention). This short follow-up time limits the study findings to the short-term only.

Given the promising results, there needs to be further evaluation of the effectiveness of the intervention. This can be done gradually by starting with a simpler research design (eg, nonrandomized control trial) to a more complex research design (eg, RCT), depending on the feasibility, timeframe, and available expertise. Additionally, it would be more informative to assess the effects of the webinar intervention with longer follow-up (eg, 3 months). Given that this is a universal intervention, the intervention can be compared with other forms of universal interventions in the workplace as well as with a wait-list control group. As this was a complex intervention, further investigations are also needed in terms of understanding the active ingredients and change processes through a mediation and mediator analysis, as well as assessing the cost-effectiveness. These figures can then be compared with those for other face-to-face or internet interventions.

As this intervention is designed for employees within the workplace, it is also important to consider the study design possibilities bearing in mind the hierarchical nature of the organization, in which employees can be nested in teams and departments within the organization’s organizational structure. In relation to this, future trials may consider cluster randomization as opposed to individual randomization considering that each employee is usually attached to the work structure of their respective teams or departments or organizations. Additionally, future trials need to also include procedures and time for recruiting at the organization and individual levels, which involved identifying and recruiting interested organizations, obtaining organizational permission and agreement, on-site technical readiness assessment, and introductory meetings as well as recruiting employees.

### Conclusions

This proof-of-concept study provides preliminary evidence that self-confidence webinars can be a potentially feasible, effective, and acceptable intervention for depression in the workplace. The webinar intervention appears to be feasible as indicated by the successful recruitment of employees, the webinar sessions successfully running, relatively low dropout, and high completion rates as well as no major technical issues. The outcome analysis reported a lower level of depression, anxiety, absenteeism, and presenteeism as well as improved self-esteem and coping flexibility among employees postintervention and 1 month after completion of the intervention. It has also been shown to be acceptable.

In addition to its potential effectiveness in an RCT, the self-confidence webinar intervention is likely to be acceptable among employees in the workplace. Apart from its accessibility advantage, the webinar offers an interactive environment that may not be possible in other technology-mediated or face-to-face interventions. The webinar was able to maintain some elements of face-to-face intervention while utilizing economical delivery and technological features that were available. This may serve as an alternative and fruitful way of reaching depressed people in the workplace.

## Appendix

Multimedia Appendix 1 Screenshots during the webinar sessions.

Multimedia Appendix 2 Example quotes from the feedback corresponding to each category.

## Abbreviations

BDI-II: Beck Depression Inventory II
CBT: cognitive behavior therapy
RCI: Reliable Change Index
RCT: randomized controlled trial
SC: stress control
UEQ: User Experience Questionnaire
