# Supplementary material for: Evaluating the Feasibility of an Innovative Self-Confidence Webinar Intervention for Depression in the Workplace: A Proof-of-Concept Study
Source: JMIR Ment Health. 2019 Apr 26;6(4):e11401. doi: 10.2196/11401 (PMC6658313; doi:10.2196/11401)
Supplement: Multimedia Appendix 1 [file mental_v6i4e11401_app1.pdf]

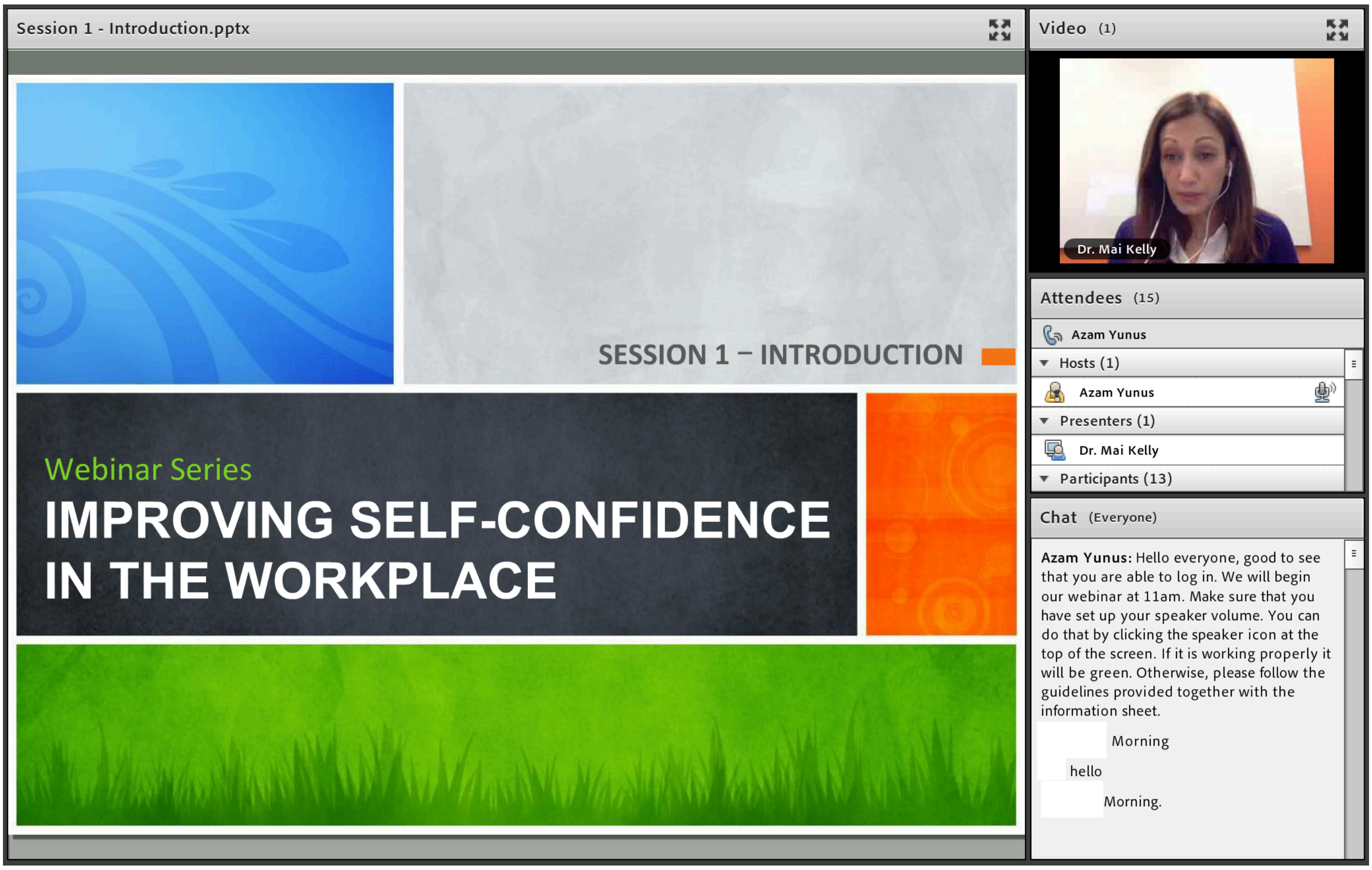
Session 1 - Introduction.pptx
SESSION 1 – INTRODUCTION
Webinar Series
IMPROVING SELF-CONFIDENCE IN THE WORKPLACE
Video (1)
Dr. Mai Kelly
Attendees (15)
Azam Yunus
Hosts (1)
Azam Yunus
Presenters (1)
Dr. Mai Kelly
Participants (13)
Chat (Everyone)
Azam Yunus: Hello everyone, good to see that you are able to log in. We will begin our webinar at 11am. Make sure that you have set up your speaker volume. You can do that by clicking the speaker icon at the top of the screen. If it is working properly it will be green. Otherwise, please follow the guidelines provided together with the information sheet.
Morning
hello
Morning.

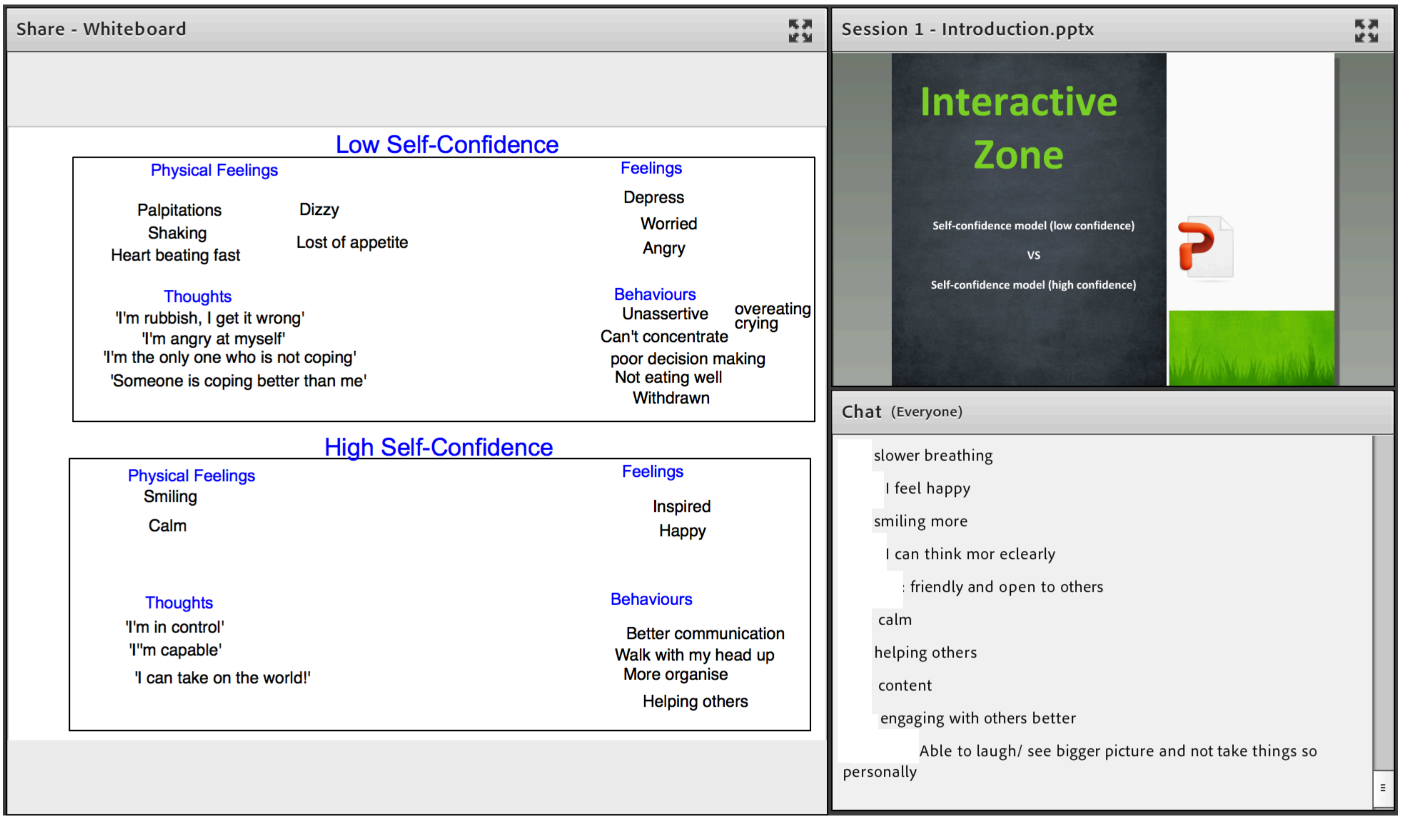
Share - Whiteboard
Low Self-Confidence
Physical Feelings
Palpitations
Shaking
Heart beating fast
Dizzy
Lost of appetite
Feelings
Depress
Worried
Angry
Thoughts
'I'm rubbish, I get it wrong'
'I'm angry at myself'
'I'm the only one who is not coping'
'Someone is coping better than me'
Behaviours
Unassertive
overeating
crying
Can't concentrate
poor decision making
Not eating well
Withdrawn
High Self-Confidence
Physical Feelings
Smiling
Calm
Feelings
Inspired
Happy
Thoughts
'I'm in control'
'I"m capable'
'I can take on the world!'
Behaviours
Better communication
Walk with my head up
More organise
Helping others
Session 1 - Introduction.pptx
Interactive Zone
Self-confidence model (low confidence)
vs
Self-confidence model (high confidence)
Chat (Everyone)
slower breathing
I feel happy
smiling more
I can think mor eclearly
: friendly and open to others
calm
helping others
content
engaging with others better
Able to laugh/ see bigger picture and not take things so personally

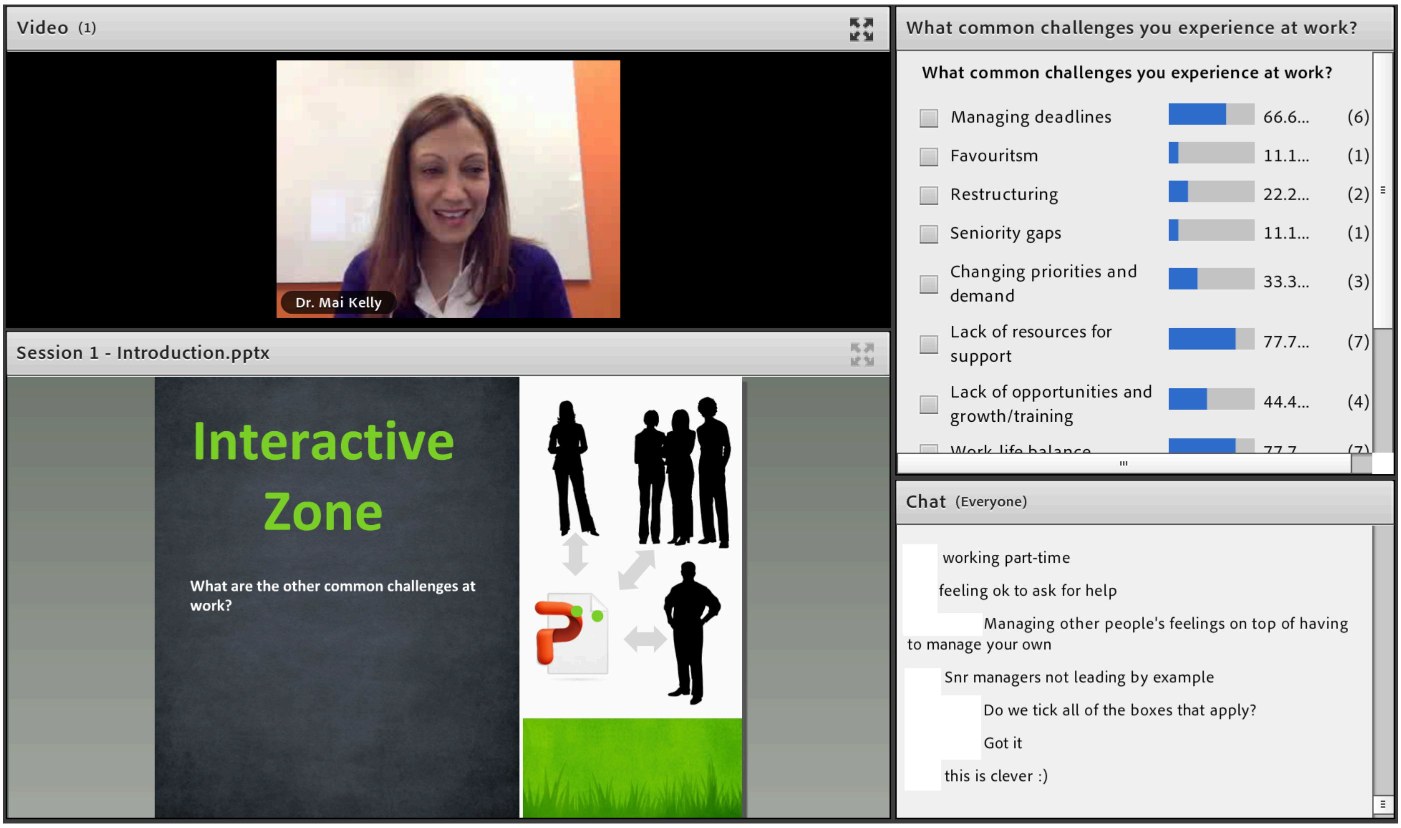
Video (1)
Dr. Mai Kelly
Session 1 - Introduction.pptx
Interactive Zone
What are the other common challenges at work?
What common challenges you experience at work?
What common challenges you experience at work?
Managing deadlines 66.6... (6)
Favouritsm 11.1... (1)
Restructuring 22.2... (2)
Seniority gaps 11.1... (1)
Changing priorities and demand 33.3... (3)
Lack of resources for support 77.7... (7)
Lack of opportunities and growth/training 44.4... (4)
Chat (Everyone)
working part-time
feeling ok to ask for help
Managing other people's feelings on top of having to manage your own
Snr managers not leading by example
Do we tick all of the boxes that apply?
Got it
this is clever :)

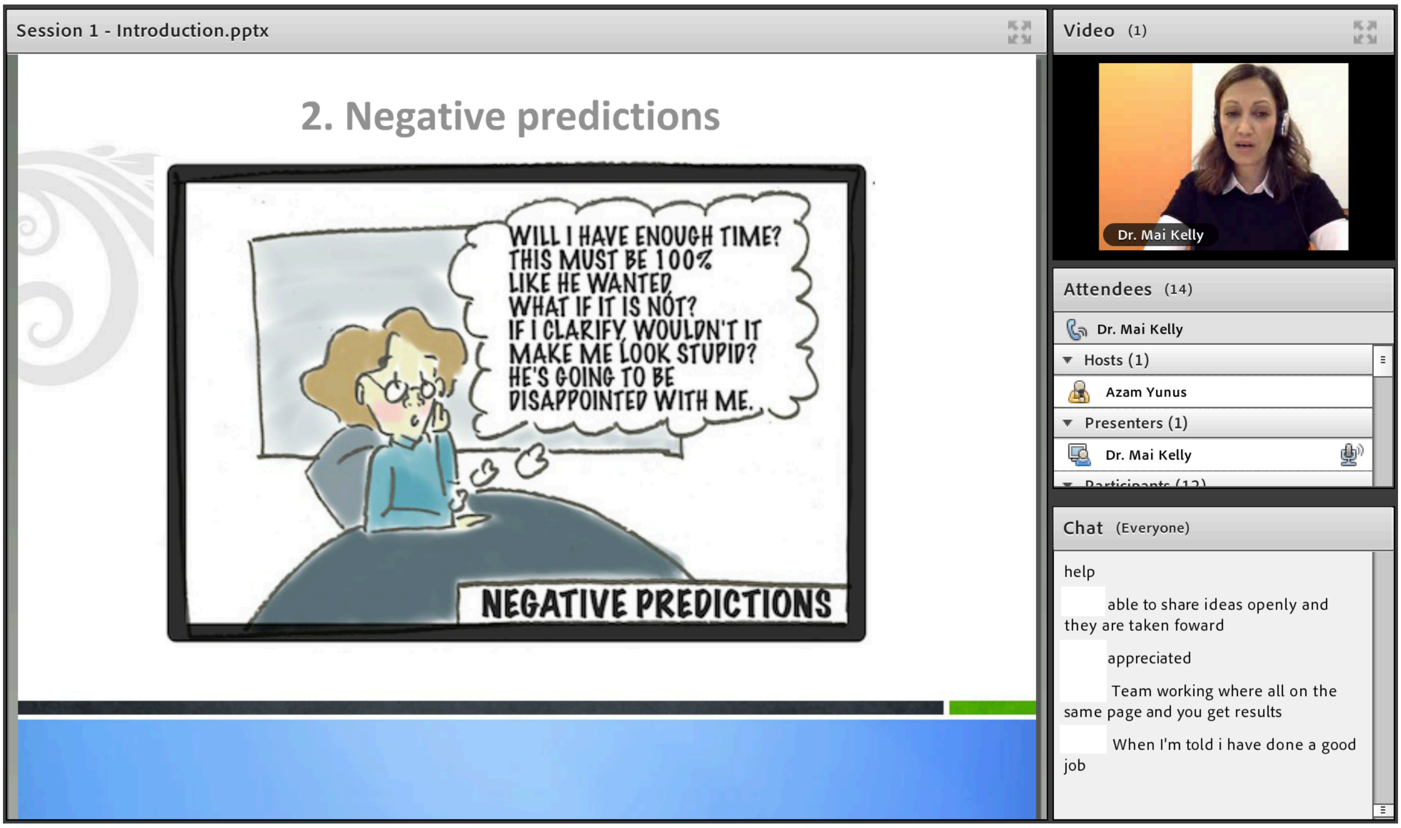
Session 1 - Introduction.pptx
2. Negative predictions
WILL I HAVE ENOUGH TIME? THIS MUST BE 100% LIKE HE WANTED. WHAT IF IT IS NOT? IF I CLARIFY, WOULDN'T IT MAKE ME LOOK STUPID? HE'S GOING TO BE DISAPPOINTED WITH ME.
NEGATIVE PREDICTIONS
Video (1)
Dr. Mai Kelly
Attendees (14)
Dr. Mai Kelly
Hosts (1)
Azam Yunus
Presenters (1)
Dr. Mai Kelly
Chat (Everyone)
help
able to share ideas openly and they are taken foward
appreciated
Team working where all on the same page and you get results
When I'm told i have done a good job

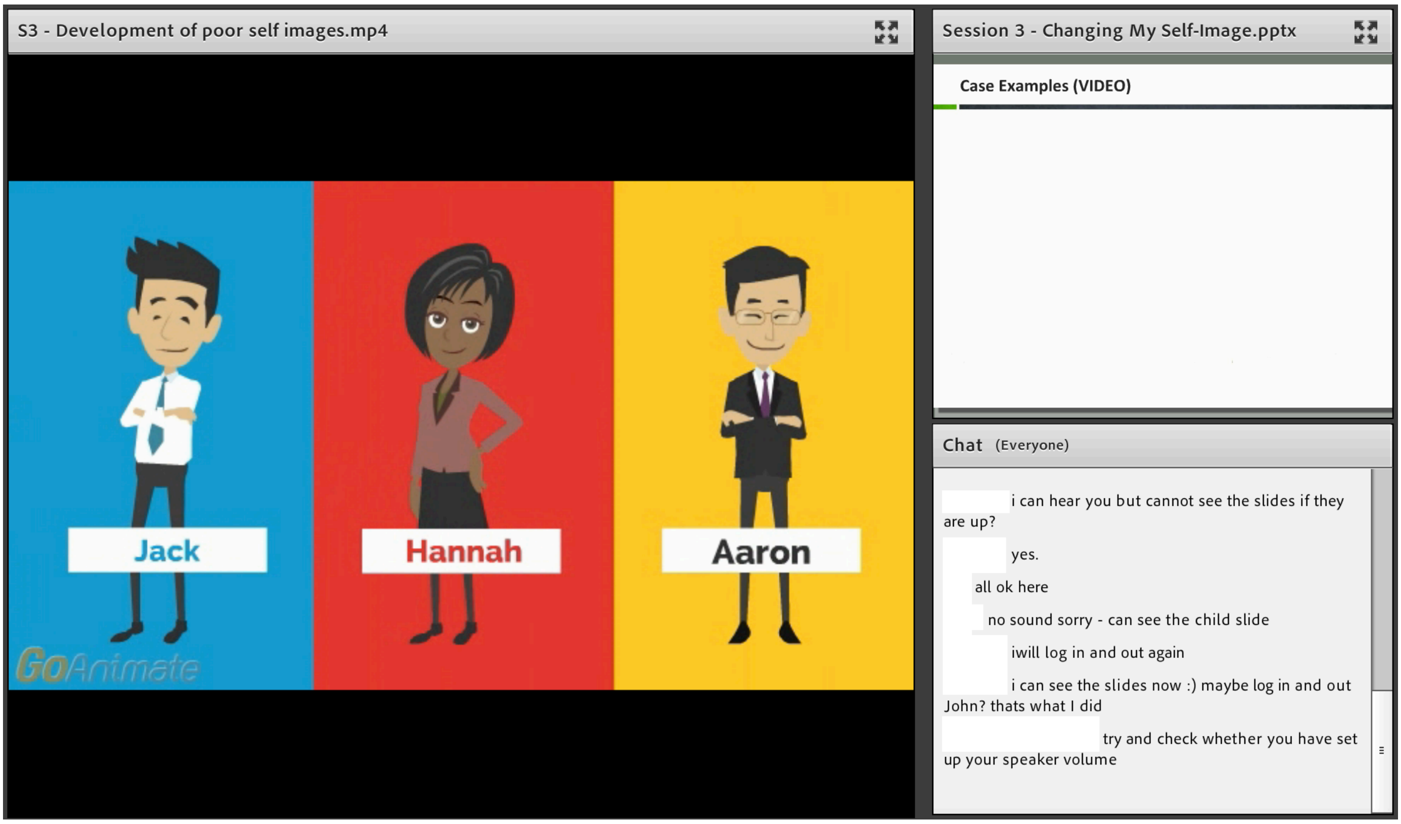
S3 - Development of poor self images.mp4
Jack
Hannah
Aaron
GoAnimate
Session 3 - Changing My Self-Image.pptx
Case Examples (VIDEO)
Chat (Everyone)
i can hear you but cannot see the slides if they are up?
yes.
all ok here
no sound sorry - can see the child slide
iwill log in and out again
i can see the slides now :) maybe log in and out John? thats what I did
try and check whether you have set up your speaker volume

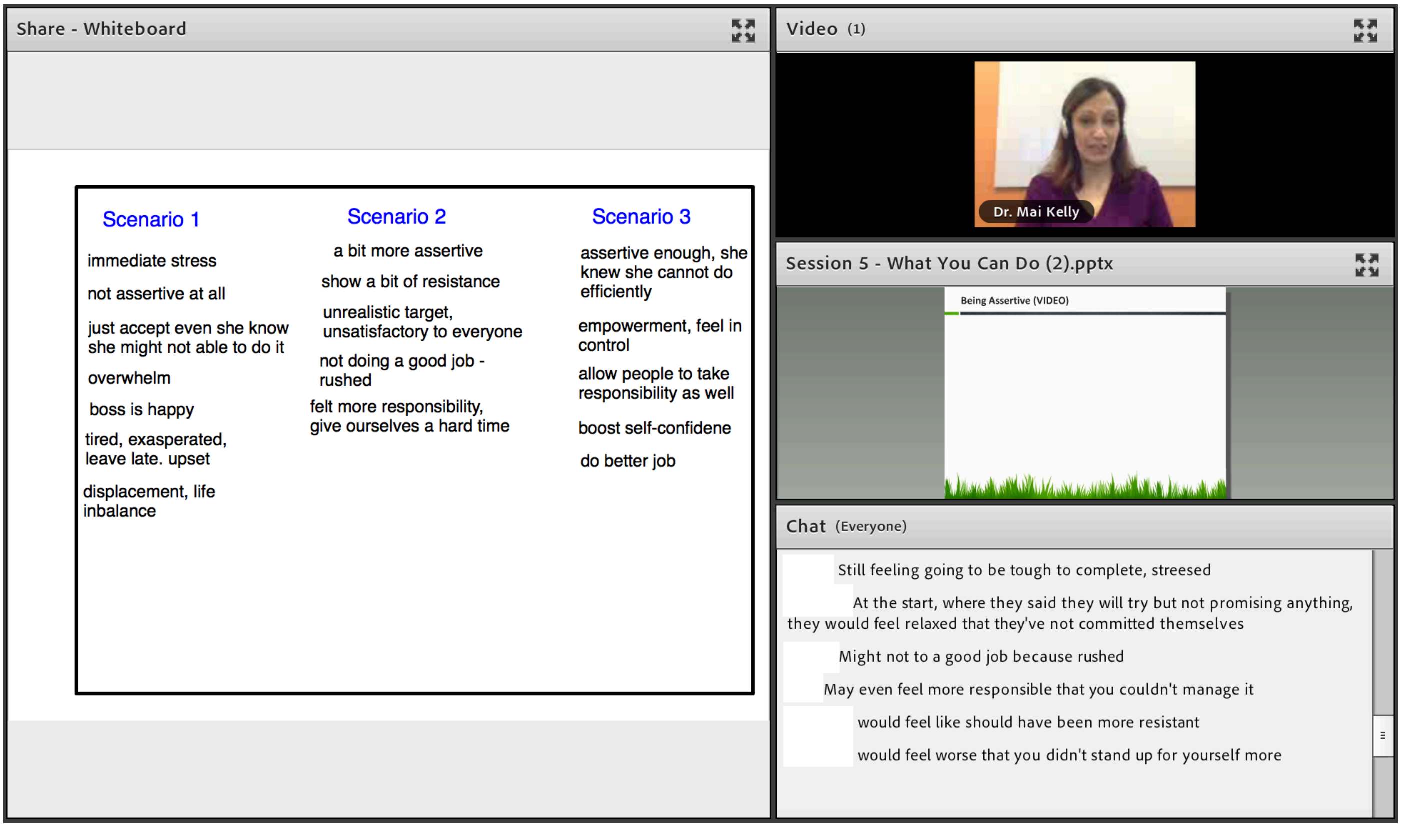
Share - Whiteboard
Scenario 1
immediate stress
not assertive at all
just accept even she know she might not able to do it
overwhelm
boss is happy
tired, exasperated, leave late. upset
displacement, life inbalance
Scenario 2
a bit more assertive
show a bit of resistance
unrealistic target, unsatisfactory to everyone
not doing a good job - rushed
felt more responsibility, give ourselves a hard time
Scenario 3
assertive enough, she knew she cannot do efficiently
empowerment, feel in control
allow people to take responsibility as well
boost self-confidene
do better job
Video (1)
Dr. Mai Kelly
Session 5 - What You Can Do (2).pptx
Being Assertive (VIDEO)
Chat (Everyone)
Still feeling going to be tough to complete, streesed
At the start, where they said they will try but not promising anything, they would feel relaxed that they've not committed themselves
Might not to a good job because rushed
May even feel more responsible that you couldn't manage it
would feel like should have been more resistant
would feel worse that you didn't stand up for yourself more

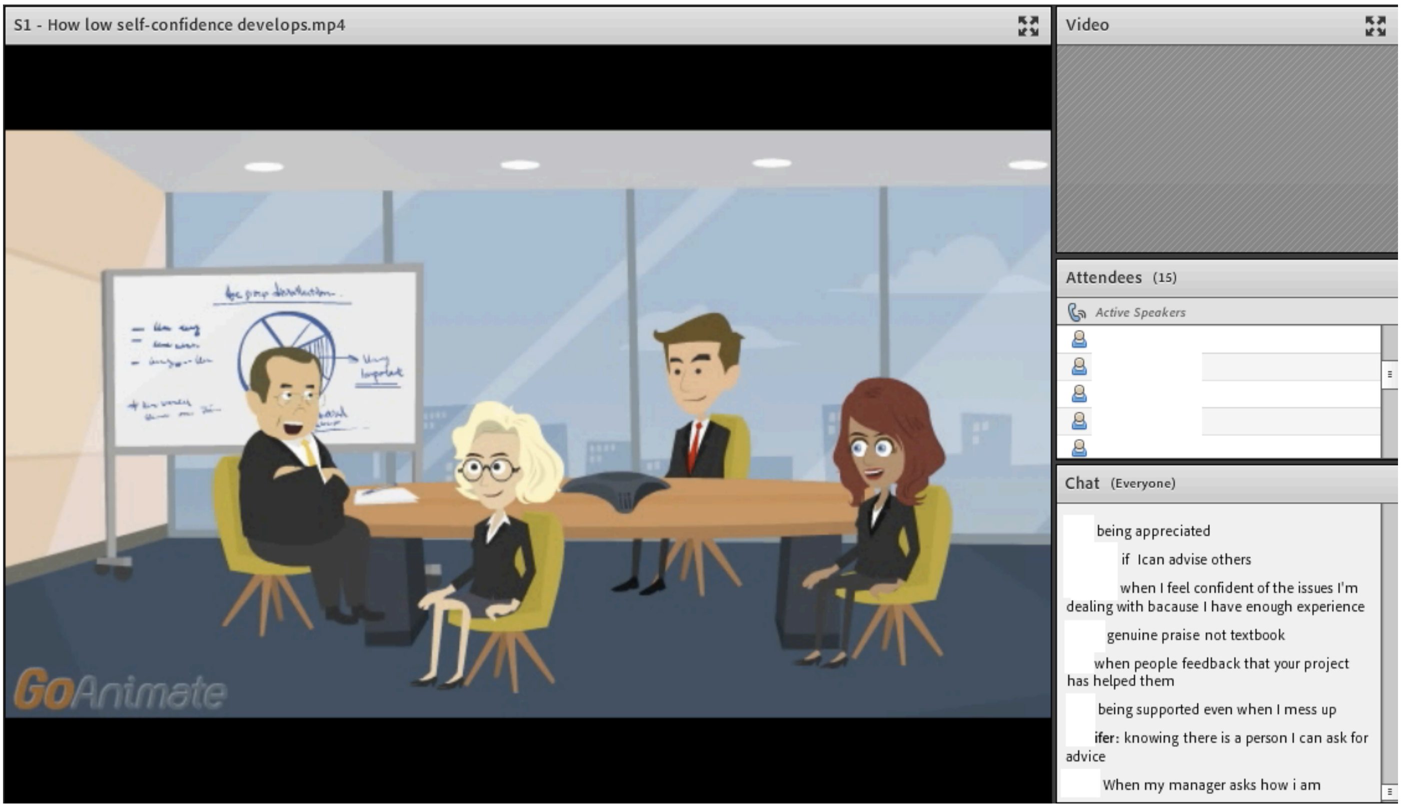
S1 - How low self-confidence develops.mp4
GoAnimate
Video
Attendees (15)
Active Speakers
Chat (Everyone)
being appreciated
if Ican advise others
when I feel confident of the issues I'm dealing with bacause I have enough experience
genuine praise not textbook
when people feedback that your project has helped them
being supported even when I mess up
ifer: knowing there is a person I can ask for advice
When my manager asks how i am

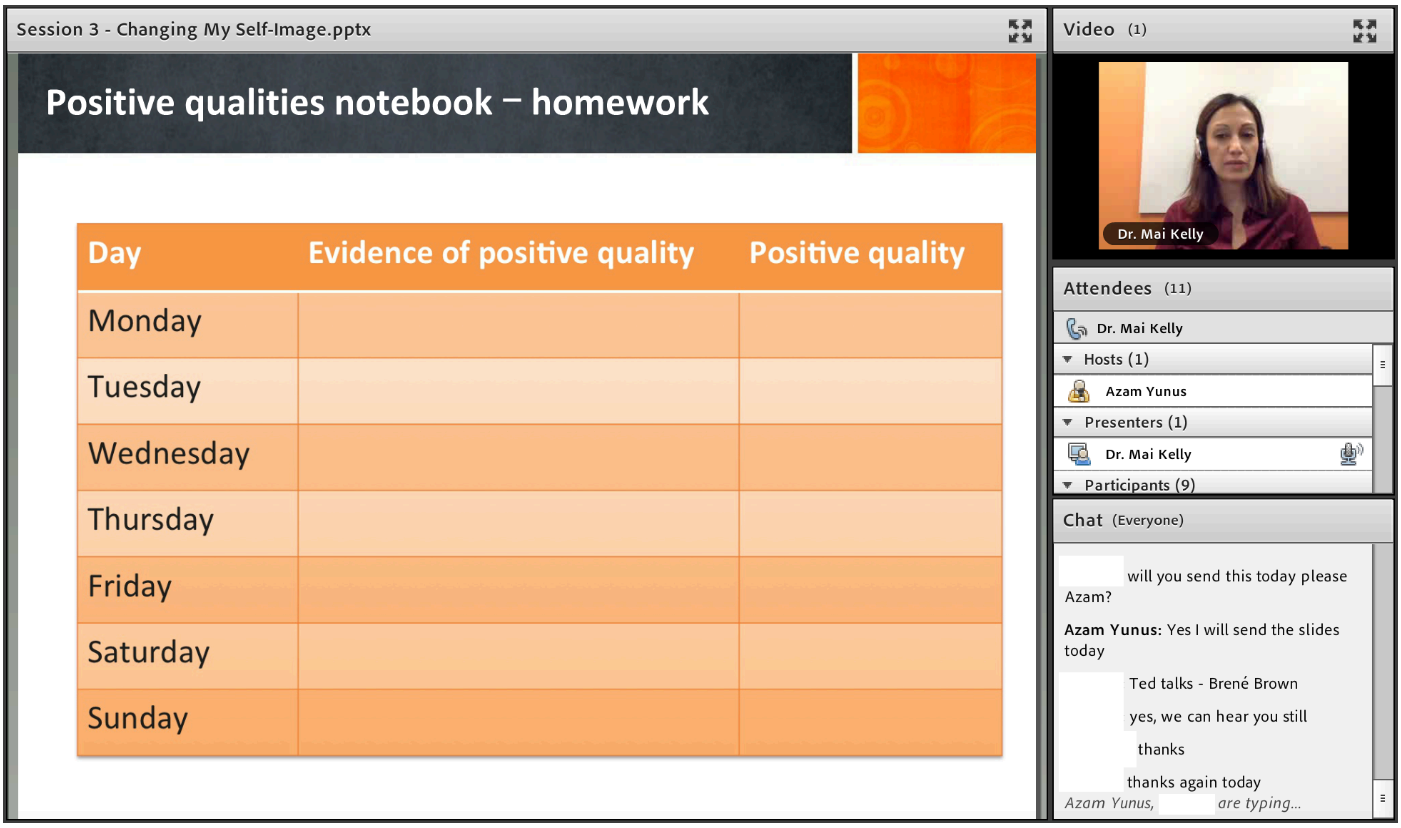
Session 3 - Changing My Self-Image.pptx
Positive qualities notebook – homework
Day
Evidence of positive quality
Positive quality
Monday
Tuesday
Wednesday
Thursday
Friday
Saturday
Sunday
Video (1)
Dr. Mai Kelly
Attendees (11)
Dr. Mai Kelly
Hosts (1)
Azam Yunus
Presenters (1)
Dr. Mai Kelly
Participants (9)
Chat (Everyone)
will you send this today please Azam?
Azam Yunus: Yes I will send the slides today
Ted talks - Brené Brown
yes, we can hear you still
thanks
thanks again today
Azam Yunus, are typing...
