# Supplementary material for: Evaluating the Feasibility of an Innovative Self-Confidence Webinar Intervention for Depression in the Workplace: A Proof-of-Concept Study
Source: JMIR Ment Health. 2019 Apr 26;6(4):e11401. doi: 10.2196/11401 (PMC6658313; doi:10.2196/11401)
Supplement: Multimedia Appendix 2 [file mental_v6i4e11401_app2.pdf]

Table 8: Facilitator sub-categories and examples of quotes

**Facilitators**	**Frequency (%)**	**Example quotes**
Content	12 (26)	*'Sometimes you have to take in so much during an intensive days learning course, this one was a good example of the benefits of doing a modular approach.'(R4)*
		'The mix of different methods used livened the webinars up and made them interactive yet informative.' (R16)
Interactive Zones	12 (26)	*'The way it was delivered was good as it allowed people to share thoughts /ideas/concerns that they may not have done if the course was delivered in person.' (R2)*
		'Interactive sessions worked well together to bring the context / topic to life.' (R4)
Usefulness	9 (19)	*'I found the short course very helpful and beneficial to me so it would be interesting to see how much more it can help as I am a nervous wreck most of the time.' (R24)*
		'I found it valuable and can already see ways that it is making a difference in both my personal and professional life.' (R4)
Image/Comic/ videos	4 (9)	*'Interactive practices were engaging and the videos of examples helpful.' (R16)*
		'Liked being able to see and hear the presenter.' (R14)
Resources	4 (9)	*'Great to be sent slides/recordings after webinar.' (R20)*
		'Liked the emails prior to each session, notes being forwarded post session.' (R6)
Presenter's skill	3 (6)	*'The presenter was very compassionate and sensitive to what was being said which is good.' (R15)*
		'The way the host noted our comments was good.' (R8)
Homework	1 (2)	*'I found the homework really beneficial.' (R5)*
Timing/duration	1 (2)	*'Having the course spread out across a 6 week (one hour blocks) period was valuable as it gave me the opportunity to reflect on each of the pockets of learning.' (R4)*
IT capability	1 (2)	*'Good format and easy to access.' (R8)*

Table 9: Barrier sub-categories and examples of quotes

**Barriers**	**Frequency (%)**	**Example quotes**
Animation videos	4 (36)	*'Change the monotone voices used in the plays! it felt like it brings the message behind it to a less important level it was hard to take it seriously sometimes because I was concentrating more on their voices than the message for longer than I needed to.' (R15)*
		'The cartoon videos weren't great - more real life examples and practical tips would be useful.' (R8)
Timing	2 (18)	*'I can't really think about how the training could be improved except more available times to attend it as I struggled to attend each session live every week.' (R11)*
		'Make sure client has free time to do this.' (R18)
Commitment	2 (18)	*'Unfortunately due to work commitments I was only able to join a few sessions so don't really feel I can comment on the programme.' (R10)*
		'I was hoping that I could have gained more from the sessions but my work got increasingly more demanding and I have not benefitted as much as I had hoped.' (R11)
Duration	1 (9)	*'I think the course was just a few sessions too long the course could have been compacted and been more to the point of dealing with stress and managing our self-confidence'(R2)*
IT issues	1 (9)	*'Slight technical hitches could be ironed out.' (R20)*
Homework	1 (9)	*'I was not very good at doing any homework but can see how this would be useful for some who were experiencing problems.' (R3)*

Table 10: Suggestions sub-categories and examples of quotes

**Suggestions**	**Frequency (%)**	**Example quotes**
Resources	4 (31)	*'Send everyone all of the links to video recordings at the end of the 6 sessions.' (R19)*
		'Perhaps a small resource at the end of each session would be helpful?' (R15)
Continuity	4 (31)	*'Would have been nice to join a monthly group in workplace or to have continued indefinitely as helped me make some progress with colleagues/my own self-image in the workplace.' (R14)*
		'Other colleagues are interested in this webinar - will you be running it again?' (R7)
Content	2 (15)	*'Would be good to focus on strategies to deal with different characters and personalities as that often is a factor in how confident you feel in the workplace.' (R1)*
		'Stress coping mechanisms because everyone in some shape or form struggles with self-confidence but some people deal with it better. It's the dealing and managing the confidence that matters.' (R2)
Timing/Duration	1 (8)	*'It would be great if this was longer and more in depth.' (R24)*
Homework	1 (8)	*'Maybe a bit more discussion of the homework once done, any challenges etc.' (R20)*
IT capability	1 (8)	*'It will always be an issue. Perhaps acknowledge this with all participants before the next webinar and state that in past sessions, staff has not been able to hear/see however when they log in again it does work etc..' (R15)*

Table 11: General comment sub-categories and examples of quotes

**Suggestions**	**Frequency (%)**	**Example quotes**
Good in general	8 (40)	*'I liked all of it!' (R14)*
		'I think the webinar ran really well, it was smooth learning journey, and everything was explained in plain English so I understood how to go away and try to apply a new technique, which I am continuing to do to this day' (R5)
Neutral	7 (35)	*'None'(R22)*
Appreciation	3 (15)	*'Thank you very much for making this available to me.' (R19)*
		'Just a big thank you, really enjoyed it!' (R20)
Negative	2 (10)	*'Some feedback questions either repeated themselves too much or the reasoning for answer not related to being stressed: e.g. wobbliness and not having enough energy due to my current physical injury.' (R17)*
		'Answering the questions in this questionnaire has made me realise that I am probably more stressed and exhausted now than when I started these sessions.' (R11)
